# A bone substitute with high affinity for vitamin D-binding protein―relationship with niche of osteoclasts

**DOI:** 10.1111/jcmm.12180

**Published:** 2013-11-28

**Authors:** Tohru Ikeda, Michiyuki Kasai, Eri Tatsukawa, Masanobu Kamitakahara, Yasuaki Shibata, Taishi Yokoi, Takayuki K Nemoto, Koji Ioku

**Affiliations:** a Department of Oral Pathology and Bone Metabolism, Nagasaki University Graduate School of Biomedical SciencesNagasaki, Japan; b Department of Safety Research on Blood and Biological Products, National Institute of Infectious DiseasesTokyo, Japan; c Graduate School of Environmental Studies, Tohoku UniversitySendai, Japan; d Department of Molecular Biology, Nagasaki University Graduate School of Biomedical SciencesNagasaki, Japan; e Division of Experimental Immunology Institute for Genome Research, University of TokushimaTokushima, Japan; f Department of Chemistry Faculty of Economics, Keio UniversityYokohama, Japan

**Keywords:** hydroxyapatite, osteoclast, vitamin D-binding protein, macrophage activating factor, serum proteins

## Abstract

The biological activity of osteoblasts and osteoclasts is regulated not only by hormones but also by local growth factors, which are expressed in neighbouring cells or included in bone matrix. Previously, we developed hydroxyapatite (HA) composed of rod-shaped particles using applied hydrothermal methods (HHA), and it revealed mild biodegradability and potent osteoclast homing activity. Here, we compared serum proteins adsorbed to HHA with those adsorbed to conventional HA composed of globular-shaped particles (CHA). The two ceramics adsorbed serum albumin and γ-globulin to similar extents, but affinity for γ-globulin was much greater than that to serum albumin. The chemotactic activity for macrophages of serum proteins adsorbed to HHA was significantly higher than that of serum proteins adsorbed to CHA. Quantitative proteomic analysis of adsorbed serum proteins revealed preferential binding of vitamin D-binding protein (DBP) and complements C3 and C4B with HHA. When implanted with the femur of 8-week-old rats, HHA contained significantly larger amount of DBP than CHA. The biological activity of DBP was analysed and it was found that the chemotactic activity for macrophages was weak. However, DBP-macrophage activating factor, which is generated by the digestion of sugar chains of DBP, stimulated osteoclastogenesis. These results confirm that the microstructure of hydroxyapatite largely affects the affinity for serum proteins, and suggest that DBP preferentially adsorbed to HA composed of rod-shaped particles influences its potent osteoclast homing activity and local bone metabolism.

## Introduction

Bone metabolism is strictly regulated by the balance of osteogenic activity of osteoblasts and bone resorbing activity of osteoclasts. The biological activities of osteoblasts and osteoclasts are regulated not only by hormones but also by local factors. The importance of hormonal regulation of bone metabolism is obvious considering symptoms of diseases like hypercalcaemia and osteopenia caused by hyperparathyroidism and osteoporosis caused by oestrogen deficiency in post-menopause 1,2. In addition to hormonal regulation, local regulation has also been found to be very important to bone metabolism. Potent osteogenic factors, bone morphogenetic proteins (BMPs), were initially isolated from bone matrix proteins.

Interestingly, osteoblasts in turn express one of the most important osteoclastogenic factors, receptor activator of nuclear factor kappa-B ligand (RANKL). When RANKL binds to its receptor RANK, which is expressed in osteoclast progenitor cells, osteoclast progenitor cells differentiate into osteoclasts 4. In addition to the coupling of bone formation to resorption described above, many types of interconnected cell biological regulation have been shown to contribute to regulate bone metabolism 5–9. Hence, study of the local regulation of bone metabolism in regions implanted with a bone substitute is very important.

Previously, we developed a unique calcium-deficient hydroxyapatite (HA) composed of rod-shaped particles using applied hydrothermal methods 10. We implanted a cylindrical block of HA composed of rod-shaped particles prepared by applied hydrothermal methods (HHA) and HA synthesized by conventional sintering (CHA) into rabbit femurs and compared their biological behaviours. Surprisingly, HHA exhibited biodegradability in contrast to CHA, which was almost unbiodegradable during 24 weeks after implantation. It was striking that bone volume/tissue volume (BV/TV) and number of osteoclasts/bone perimeter (N.Oc/B.Pm) were significantly larger in the regions implanted with HHA than those implanted with CHA 11. These findings were confirmed in a rat implantation model using spherical granules of HHA and CHA. In addition, stimulation of osteogenesis was suggested to be associated with osteoclasts from the finding that culture supernatants of osteoclasts stimulated the differentiation of osteoblasts 12.

Considering these findings, potent osteoclast homing activity of HHA might be associated with larger BV/TV in regions implanted with HHA than those implanted with CHA. However, the mechanism of potent osteoclast homing activity of HHA remains unclear. The biodegradable nature of HHA is thought to increase the local concentration of calcium and/or phosphate, and it may affect local bone metabolism through regulation of the biological activity of osteoblasts and osteoclasts as suggested previously 13–16. Hence, high turnover of bone metabolism in the region implanted with HHA, which we reported previously, might be associated with the biodegradation of HHA 17. Surface roughness of implant materials has been reported to stimulate osteogenesis 18–19, and a rod-shaped microstructure might also be directly associated with osteogenesis or osteoclastogenesis in the implanted region. Considering the importance of bone matrix proteins included in the bone-to-bone metabolism by their release from bone upon osteoclastic bone resorption, there is also a possibility that proteins adsorbed to implanted porous bone substitutes affect local bone metabolism. In this study, the mechanism of potent osteoclast homing activity of HHA was analysed by characterizing its affinity for serum proteins. The biological activity of serum protein preferentially adsorbed to HHA was also analysed.

## Materials and methods

### Preparation of ceramics

Alpha-tricalcium phosphate (α-TCP) powder (Taihei Chemical Ind. Co. Ltd., Osaka, Japan) was mixed and kneaded with 10% gelatine solution, and dropped into a stirred oil bath heated at 80°C. The bath was then chilled on ice and spherical α-TCP/gelatine granules were formed. The granules were separated from the oil, rinsed and sintered at 1200°C for 10 min. to remove the gelatine and to maintain the crystal phase of α-TCP. The formed α-TCP granules were set in an autoclave at 160°C under saturated water vapour pressure for 20 hrs 20. In an autoclave, α-TCP is reacted with water, the α-TCP dissolves into water, the supersaturation with respect to HA is achieved and HA is formed 21. Synthesized HHA granules were sieved and granules 0.5–0.6 mm in diameter were used for experiments. Spherical CHA granules were prepared by sintering at 900°C for 3 hrs from the same chemical purity grade HA powders with stoichiometric composition. The porosity of HHA and CHA granules was designed to be 70%.

### Serum protein adsorption to ceramic granules

To analyse the optimum conditions of adsorption and elution of serum proteins, standard procedures for HA column chromatography were used 22,23. Four hundred mg of HHA or CHA granules was inserted into an Econo-Column^®^ (Bio-Rad, Hercules, CA, USA), equilibrated with 0.05 M sodium phosphate buffer (pH. 6.8), and 12 mg of human γ-globulin (Keketsuken, Kumamoto, Japan) or bovine serum albumin (BSA; MP Biomedicals, Aurora, OH, USA) dissolved in 2 ml of 0.05 M sodium phosphate buffer was applied. Each column was washed with 3 ml of 0.05 M sodium phosphate buffer three times, and bound materials were eluted with 2 ml each of 0.05 M sodium phosphate buffer with stepwise increase in the concentration of NaCl (0.2, 0.4, 0.6, 0.8 and 1.0 M). The other columns, each of which contained HHA or CHA granules, were eluted with 2 ml each of 0.5 M sodium phosphate buffer (pH. 6.8) followed by 0.5 M sodium phosphate buffer with stepwise increase in the concentration of NaCl (0.2, 0.4, 0.6 and 0.8 M).

The optimum time for adsorption of serum proteins was analysed as follows. One hundred mg of HHA or CHA granules was inserted into a 2 ml microcentrifuge tube, equilibrated with 0.05 M sodium phosphate buffer at 4°C overnight, and 50 μl of normal human serum (Infectrol^®^ E-00; Kyowa Medex Co., Tokyo, Japan) diluted with 450 μl of 0.05 M sodium phosphate buffer was applied and adsorbed with gentle shaking at 4°C for 1, 4, 12, 24 and 48 hrs. After each period of adsorption, ceramic granules were washed with 1 ml of 0.05 M sodium phosphate buffer three times for 5 min. each with gentle shaking at 4°C, then eluted with 0.5 ml of 0.5 M sodium phosphate buffer for 1 hr with gentle shaking at 4°C. Protein quantification was performed by colorimetry using a Pierce^®^ BCA Protein Assay Kit (Thermo Scientific, Rockford, IL, USA).

Eluates for chemotactic assay and quantitative proteomic analysis were prepared as follows. Six hundred mg of each type of ceramic granule was divided into three samples of 200 mg of granules, and each of these samples was inserted into a plastic tube (Falcon 2063; BD Falcon Labware, Franklin Lakes, NJ, USA) and equilibrated with 0.05 M sodium phosphate buffer at 4°C overnight. The next day, the buffer was discarded, and 200 μl of normal human serum (Infectrol^®^ E-00; Kyowa Medex Co.) diluted with 200 μl of 0.05 M sodium phosphate buffer was applied to the ceramic granules in each plastic tube and adsorbed at 4°C for 24 hrs with gentle shaking. The supernatant was removed and ceramic granules were washed with 3 ml of 0.05 M sodium phosphate buffer three times for 5 min. each with gentle shaking at 4°C, then eluted with 0.5 ml of 0.5 M sodium phosphate buffer for 10 min. with gentle shaking at 4°C. Part of each eluate was used for protein quantification and the remainder was combined for each kind of ceramic and dialyzed in PBS using Slide-A-Lyzer^®^ Dialysis Cassettes (Thermo Scientific) following the manufacturer’s instructions. Part of the dialyzed sample was used for protein quantification and the remainder was used for chemotactic assay and quantitative proteomic analysis. For chemotactic assay, each eluate was adjusted to a protein concentration of 240 μg/ml. Quantitative proteomic analysis was performed with an iTRAQ^®^ system, which was consigned to Filgen Inc., Nagoya, Japan. Normal mouse serum (Nippon SLC, Fukuoka, Japan) was adsorbed, eluted and dialyzed as described above, and used for SDS-PAGE analysis.

### Chemotactic assay

Mouse bone marrow macrophages prepared from femurs and tibiae of 5-week-old female ddY mice were expanded *in vitro* in α-minimum essential medium (Sigma-Aldrich, St. Louis, MO, USA) supplemented with 10% foetal bovine serum and 30 ng/ml macrophage colony-stimulating factor (M-CSF; Sigma-Aldrich) as described previously 12. 1 × 10^5^ macrophages suspended in 400 μl of serum-free α-minimum essential medium supplemented with 30 ng/ml M-CSF were added to a chamber (Chemotaxicell 5 μm pore size; Kurabo, Osaka, Japan). The chamber was immediately inserted into a 24-type well with 800 μl of the serum-free culture medium supplemented with 30 ng/ml M-CSF and an eluate from HHA or CHA granules adsorbed to normal human serum. The volume of an eluate added to the culture medium was adjusted to 80 μl. Chemotactic assay supplemented with M-CSF and DBP or DBP-maf in the bottom well was also performed in the same manner. As a control, 80 μl of PBS was added to the culture medium. After 8 hrs, chambers were fixed with methanol, stained with Mayer’s haematoxylin and macrophages that migrated from the 5-μm-pore-sized membrane were quantified using an industrial epi-illumination microscope (ECLIPSE LV100D; Nikon, Tokyo, Japan) equipped with a digital camera (DS-Ri1-U2; Nikon).

Data were evaluated with the *t*-test using the results of three independent experiments and a *P* < 0.05 was considered significant.

### Preparation of vitamin D-binding protein (DBP)-macrophage activating factor (DBP-maf)

Vitamin D-binding protein-maf was prepared by deglycosylation of DBP, also known as Gc-globulin (Mixed Type, Human Plasma; Merck, Darmstadt, Germany) as described previously 25. Prior to deglycosylation, β-D-galactosidase-Sepharose beads were prepared as follows. Cyanogen bromide-activated Sepharose 4B (GE Healthcare Life Sciences, Uppsala, Sweden) was washed with 1 mM HCl, followed by washing with DDW and equilibration with coupling buffer (0.1 M NaHCO_3_, 0.5 M NaCl, pH 8.3). Thousand units of β-D-galactosidase (Wako Pure Chemical Ind., Osaka, Japan) were then added to 0.5 g of equilibrated Sepharose 4B for 2 hrs at room temperature, and the following methods to prepare β-D-galactosidase-Sepharose beads were as described previously 26. The enzymatic activity of β-D-galactosidase-Sepharose beads was determined using 2-nitrophenyl-β-D-galactopyranoside (Sigma-Aldrich). DBP was deglycosylated with 0.02 U of β-D-galactosidase-Sepharose and 0.01 U of neuraminidase-agarose (Sigma-Aldrich) beads under conditions of 0.05 mg/ml in PBS (pH 6.0) with 10 mM MgCl_2_ at room temperature for 4 hrs. After removing the beads by centrifugation, supernatant was used for biological assays as DBP-maf solution.

### Quantification of adsorbed DBP

D-binding protein was adsorbed to HHA and CHA granules following the method to prepare human serum eluates for chemotactic assay and quantitative proteomic analysis as described above. Instead of 200 μl of normal human serum diluted with 200 μl of 0.05 M sodium phosphate buffer, 0.5 mg of DBP diluted in 400 μl of 0.05 M sodium phosphate buffer was applied to the ceramic granules in each plastic tube and adsorbed at 4°C for 24 hrs with gentle shaking, then eluted with 0.5 ml of 0.5 M sodium phosphate buffer for 10 min. with gentle shaking at 4°C. Protein quantification was performed with a Quantikine^®^ Human Vitamin D-Binding Protein Immunoassay kit (R&D Systems Inc., Minneapolis, MN, USA).

For *in vivo* experiments, 10 female 8-week-old Wistar rats were anaesthetized, a dead-end bone defect 2 mm in diameter and 3 mm in depth was created in the medial cortex of the right femur just proximal to the epiphyseal growth plate using a Kirschner wire. The defect was irrigated with saline, 15 mg of HTCP or CTCP granules was implanted into the defect. Five animals were used for each ceramic. Two days after the implantation, animals were killed and ceramic granules were recovered. After the washing with 0.05 M sodium phosphate buffer for 6 hrs, proteins were eluted with 0.5 M sodium phosphate buffer for 12 hrs with gentle shaking. The buffer and ceramic granules were then grinded in an agate mortar with a pestle and the supernatant was recovered after centrifugation. Protein quantification was performed by colorimetry as described above, and DBP was quantified using a Rat gc-globulin ELISA test kit (Life Diagnostics Inc., West Chester, PA, USA). Animal rearing and experiments were performed at the Biomedical Research Center, Center for Frontier Life Sciences, Nagasaki University, following the Guidelines for Animal Experimentation of Nagasaki University (Approval No. 0703010564).

### *In vitro* osteoclastogenesis

Mouse bone marrow macrophages prepared from the femora and tibiae of 5-week-old female ddY mice were expanded *in vitro* in α-minimum essential medium supplemented with 10% foetal bovine serum and 30 ng/ml M-CSF (Sigma-Aldrich). The macrophages (1 × 10^4^ cells/cm^2^) were mixed with NIH3T3 cells (1 × 10^4^ cells/cm^2^) expressing human RANKL cDNA 27 and mouse M-CSF, and seeded in 48-well plates with α-minimum essential medium supplemented with 10% foetal bovine serum and 0.01, 0.1 or 1 μg/ml DBP, DBP-maf or PBS. These culture media were changed every other day up to day 6 of the culture period. At day 7, these cultures were fixed with 4% paraformaldehyde in 0.1 M sodium phosphate buffer (pH 7.2) for 10 min. and tartrate-resistant acid phosphatase (TRAP) activity was stained using fast red RC salt (Sigma-Aldrich) as a coupler and naphthol AS-MX phosphate (Sigma-Aldrich) as a diazonium salt as described previously 27. Multinucleated cells with more than three nuclei were counted. Four 48-wells for each concentration of DBP or DBP-maf were analysed and data were evaluated with the *t*-test. *P* < 0.05 was considered significant.

## Results

### Prepared ceramic granules

**Figure 1:**
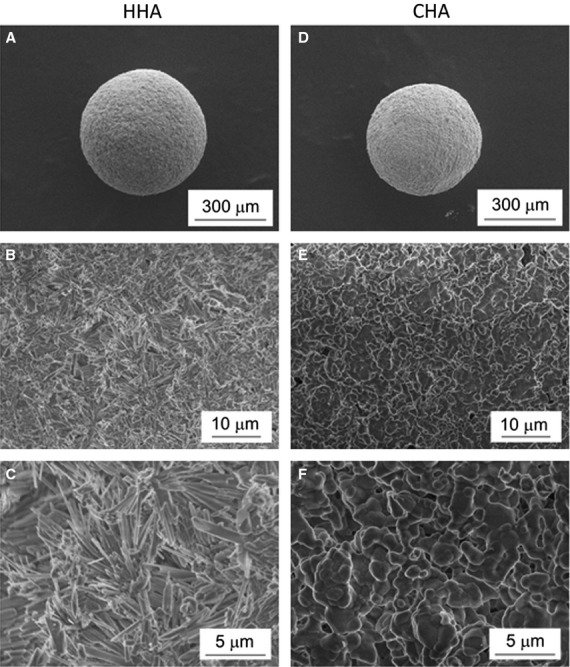
Scanning electron micrographs of the overview (A and D) and the microstructure (B, C, E and F) of HHA (A–C) and CHA (D–F) granules.

**Figure 2:**
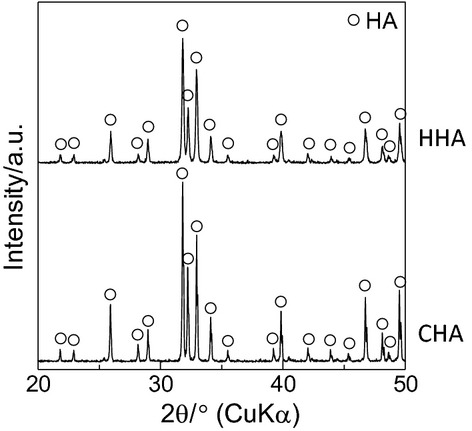
X-ray diffractometry (XRD) of implants used in this study. From XRD patterns, the main phases of HHA and CHA were assigned to HA.

Rod-shaped particles of spherical HHA granules and globular-shaped particles of spherical CHA granules were confirmed by a scanning electron microscope (SU8000; Hitachi, Ltd., Tokyo, Japan; Fig. 1). Synthesized HHA and CHA granules were analysed by powder X-ray diffractometry with graphite-monochromatized CuKα radiation, operating at 40 kV and 40 mA (XRD; RINT-2200VL; Rigaku, Tokyo, Japan). We confirmed that the main phases of HHA and CHA were HA (Fig. 2).

### Serum protein adsorption

**Figure 3:**
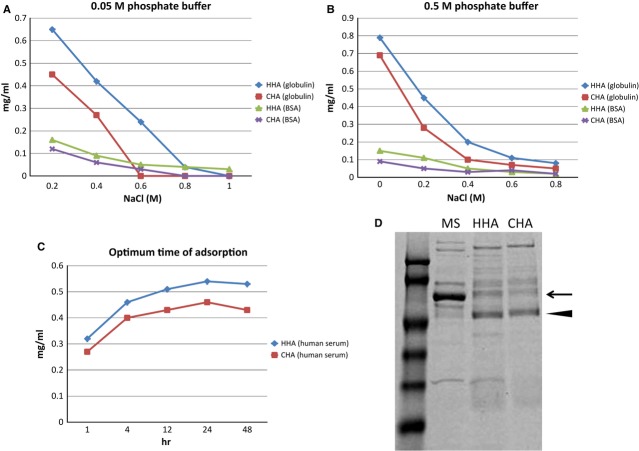
Evaluations of the adsorption of serum proteins to HHA and CHA granules. (A and B) Effects of concentrations of phosphate and NaCl. The amount of γ-globulin and BSA eluted by 0.05 M sodium phosphate buffer (A) and that eluted by 0.5 M sodium phosphate buffer (B) from 400 mg of HHA and CHA granules are shown. (C) Time-dependent variations of the amount of human serum proteins adsorbed to 100 mg of HHA and CHA granules. (D) Protein profiles from SDS-PAGE analysis of eluates of HHA and CHA adsorbed to mouse serum.

Protein concentrations in eluates with stepwise increase in the concentration of NaCl in 0.05 or 0.5 M sodium phosphate buffer were analysed. When using a 0.05 M sodium phosphate buffer, γ-globulin and BSA were largely eluted with more than 0.4 M NaCl for eluates from both HHA and CHA granules (Fig. 3A). When using a 0.5 M sodium phosphate buffer, γ-globulin and BSA were effectively eluted without NaCl, but the addition of 0.2 M NaCl was helpful (Fig. 3B). To evaluate the time-dependent efficacy of protein adsorption to ceramic granules, normal human serum was adsorbed to HHA and CHA granules for 1, 4, 12, 24 and 48 hrs. The amount of adsorbed serum proteins reached a maximum at 24 hrs. The amount of human serum proteins adsorbed to HHA was about 20% greater than that adsorbed to CHA (Fig. 3C).

Serum protein profiles of eluates from HHA and CHA were analysed by SDS-PAGE. Compared with original mouse serum, serum albumin (indicated by an arrow) had largely disappeared from both eluates, but immunoglobulin (indicated by an arrowhead) remained. Protein profiles in eluates from HHA and CHA were similar (Fig. 3D).

### Quantitative proteomic analysis

**Figure 4:**
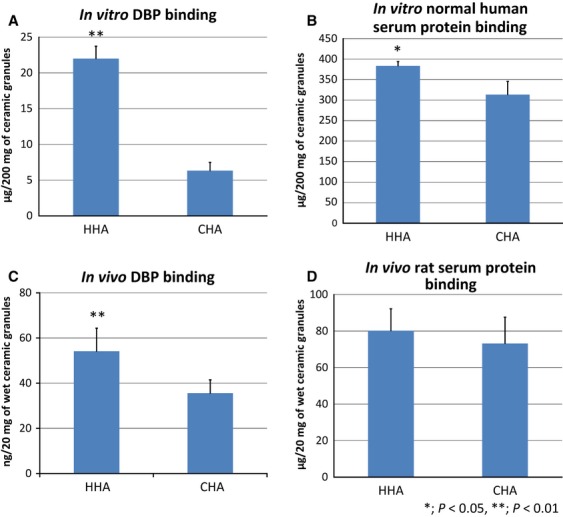
Quantification of DBP and serum proteins adsorbed to HHA and CHA. (A) DBP was adsorbed to HHA and CHA, and DBP in each eluate was quantified by ELISA. (B) Normal human serum was adsorbed to HHA and CHA, and serum protein in each eluate was quantified by colorimetry. (C and D) DBP (C) and total proteins (D) adsorbed to HHA and CHA implanted with the rat femur was quantified by ELISA and colorimetry, respectively. Twenty milligram of wet ceramic granules included ∼11 mg of dry ceramic granules. Data were evaluated using the *t*-test with the results from three individual experiments. *P < 0.05, **P < 0.01.

Serum proteins adsorbed to HHA were quantitatively compared with those adsorbed to CHA using quantitative proteomic analysis. A summary of the proteins that were adsorbed to HHA more abundantly than CHA was shown in Table 1. Among them, the serum protein that preferentially adsorbed to HHA at the highest ratio was DBP. About 19-fold DBP was adsorbed to HHA compared with CHA, and the difference was statistically significant. In addition, about 2.7-fold complement C3 and 2.1-fold complement C4B adsorbed to HHA compared with CHA. These differences were statistically significant. Except for these proteins, there were few proteins that had a strong relationship with osteoclast activity, bone metabolism or cell adhesion. The affinity for DBP was further quantified by ELISA, and about fourfold more human DBP was adsorbed to HHA than CHA (Fig. 4A). For the reference, the binding capacity of human serum proteins was also analysed. HHA adsorbed about 1.2-fold more serum proteins compared with CHA (Fig. 4B), and the result was consistent with that in Fig. 3C. The amount of DBP adsorbed to HHA implanted with the rat femur for 2 days was significantly larger than that in CHA implanted with the rat femur for 2 days (Fig. 4C), but total amount of protein adsorbed to HHA and CHA were not significantly different (Fig. 4D).

**Table 1:** Quantitative proteomic analysis of serum proteins adsorbed to HHA and CHA

Accession numbers	Protein names	Coverage (%)	Fold change HHA:CHA	*P*-values
IPI00555812.4	GC Isoform 1 of Vitamin D-binding protein	64.35	19.307	0.014
IPI00940791.1	TTR 20 kD protein	87.03	9.118	0.000
IPI00953689.1	AHSG Alpha-2-HS-glycoprotein	100.00	8.219	0.000
IPI00299435.3	APOF apolipoprotein F precursor	85.28	6.641	0.004
IPI00019581.1	F12 Coagulation factor XII	90.57	6.106	0.000
IPI00553177.1	SERPINA1 Isoform 1 of Alpha-1-antitrypsin	82.06	5.556	0.000
IPI00853525.1	APOA1 Apolipoprotein A1	91.84	4.851	0.008
IPI00942787.1	HP 42 kD protein	95.55	4.409	0.000
IPI00783987.2	C3 Complement C3 (Fragment)	91.94	2.743	0.000
IPI00418163.3	C4B complement component 4B pre-pro-protein	92.95	2.148	0.006
IPI00017601.1	CP Ceruloplasmin	73.99	1.460	0.269
IPI00021885.1	FGA Isoform 1 of Fibrinogen alpha chain	87.07	1.311	0.318
IPI00218624.1	SON Isoform I of Protein SON	90.88	1.268	0.141
IPI00749267.2	- Conserved hypothetical protein	59.14	1.265	0.080
IPI00879931.1	SERPING1 cDNA FLJ58826, highly similar to Plasma protease C1 inhibitor	97.24	1.229	
IPI00921610.1	DSPP Dentin sialophosphoprotein (Fragment)	99.72	1.154	0.060
IPI00872497.4	EI24 Etoposide-induced protein 2.4 homologue	83.29	1.152	0.535
IPI00893362.1	RYR3 Putative uncharacterized protein RYR3	88.70	1.087	0.335
IPI00759691.3	BRDT Isoform 2 of Bromodomain testis-specific protein	80.00	1.073	0.422
IPI00385250.1	PRSS3 Protease serine 4 isoform B	100.00	1.043	0.776
IPI00011769.1	NR5A2 Isoform 2 of Nuclear receptor subfamily 5 group A member 2	80.59	1.024	0.739
IPI00550906.6	CSTF2T Cleavage stimulation factor 64 kD subunit, tau variant	96.10	1.004	
IPI00945846.1	PRSS1 28 kD protein	96.9	0.9916	0.7426

*P* < 0.05 was considered to be statistically significant.

### Chemotactic activity

**Figure 5:**
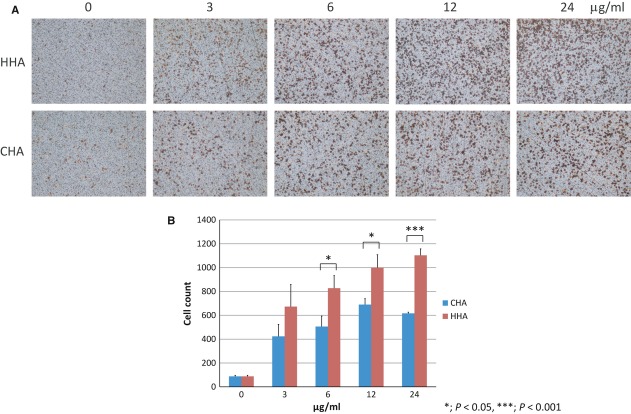
Chemotactic activity of eluates from HHA and CHA adsorbed to human serum. (A) Microscopic views of chemotactic assay using mouse bone marrow macrophages that migrated through the chamber. 0, 3, 6, 12 and 24 (μg/ml) represent final concentrations of serum proteins in eluates from HHA and CHA added to the bottom well. (B) Quantification of the number of migrated cells in chemotactic assay. *P < 0.05, ***P < 0.001.

The migration of macrophages inserted into the chambers through micropores of 5 μm in diameter was analysed at 8 hrs. With an eluate from HHA in the bottom well, the number of macrophages that migrated through micropores was significantly larger than that with an eluate of CHA in the bottom well. A dose-dependent increase in the number of migrated macrophages was detected in the experiments with eluates from HHA (Fig. 5A and B). In these experiments, the lowest protein concentration in the bottom well was 3 μg/ml and, at this concentration, the numbers of migrated macrophages with eluates of HHA and of CHA were not significantly different, although both of them were significantly larger than that of the control, in which PBS was added to the bottom well (Fig. 5B).

**Figure 6:**
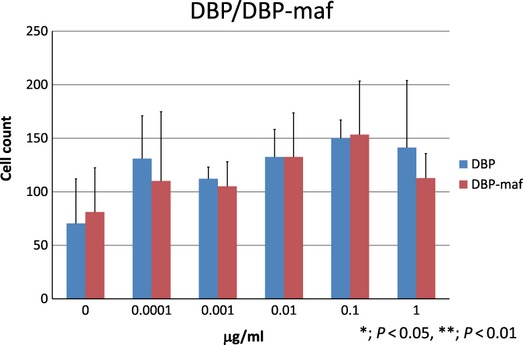
Chemotactic activity of DBP and DBP-maf. Quantification and comparison of the number of migrated cells with DBP in the bottom well and that with DBP-maf in the bottom well. 0, 0.0001, 0.001, 0.01, 0.1 and 1 (μg/ml) represent final concentrations of DBP and DBP-maf added to the bottom well. *P < 0.05, **P < 0.01.

The chemotactic activities of DBP and DBP-maf were also analysed. In the presence of 0.0001, 0.01 and 0.1 μg/ml human DBP or DBP-maf in the bottom well, chemotaxis of macrophages to the bottom was weak and no significant difference was seen between DBP and DBP-maf (Fig. 6).

### Osteoclastogenesis

**Figure 7:**
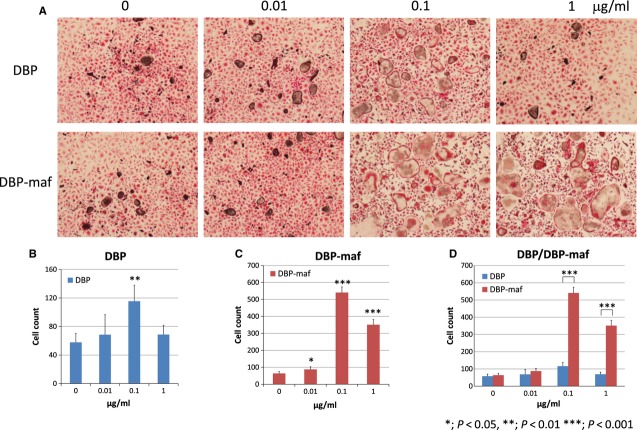
Influence of DBP and DBP-maf on osteoclastogenesis. Mouse bone marrow macrophages were cultured with RANKL-expressing fibroblasts. At day 7, cultures were fixed and TRAP activity was coloured red. 0, 0.01, 0.1 and 1 (μg/ml) represent final concentrations of DBP or DBP-maf in the culture medium. (A) Microscopic views of osteoclasts. (B and C) Quantification of osteoclasts/well treated with DBP (B) or DBP-maf (C). (D) Comparison of the number of osteoclasts treated with DBP and that treated with DBP-maf. *P < 0.05, **P < 0.01, ***P < 0.001.

The biological activities of DBP and DBP-maf on osteoclastogenesis were analysed using an *in vitro* coculture system. Without supplementation of DBP or DBP-maf, TRAP-positive cells were abundantly detected. However, the number of multinucleated osteoclasts was limited compared with that of mononuclear pre-osteoclasts. Supplementation of DBP to the culture medium revealed much less effect on osteoclastogenesis than DBP-maf. When supplemented with 0.1 or 1 μg/ml DBP-maf, the number of multinucleated osteoclasts obviously increased (Fig. 7).

## Discussion

Previously, we developed HHA using applied hydrothermal methods 10. When implanted in the bone, HHA expressed potent osteogenic and osteoclast homing activities compared with CHA 11–12. The different biological behaviour of HHA compared with that of CHA in bone promoted us to analyse the mechanism behind the difference. HA has been applied to column chromatography in biochemical fields and its affinity for proteins might affect the biological behaviour of HA in bone. HA column is widely used to isolate various kinds of proteins because of its wide range of affinity for them 22,23. Porous HA used as a bone substitute absorbs blood or extracellular fluid in the implanted region, and we analysed human serum proteins adsorbed to HHA and CHA considering an application of these ceramics to human patients. Quantitative proteomic analysis revealed notably different profiles of serum proteins adsorbed to HHA and CHA, and a larger amount of DBP was adsorbed to HHA than to CHA at the highest ratio (Table 1). DBP is one of the serum proteins that are relatively abundant. Vitamin D-binding protein-maf has a variety of biological functions, and one of its main functions is to transport vitamin D and its metabolites 28,29. Vitamin D is known to be indispensable to maintain normal bone metabolism, and DBP may have important physiological functions for bone metabolism. The binding of DBP with complement C5a was reported to enhance the chemotactic activity of C5a for neutrophils and macrophages, whereas DBP itself did not have this function 31–32. Although DBP-deficient mice did not have any apparent abnormality, *in vivo* administration of DBP-maf in osteopetrotic mice increased the population and activity of macrophages, and DBP-maf was suggested to contribute to the growth and stimulation of macrophages 33–36. Stimulation of osteoclast activity by DBP-maf was also reported using an *in vitro* culture system 37. However, this *in vitro* study was performed with isolated rat osteoclasts and the contribution of DBP and DBP-maf to osteoclastogenesis remained unclear.

In this study, the biological function of DBP, which was preferentially adsorbed to HHA and its deglycosylated form DBP-maf, on the chemotaxis of macrophages and osteoclastogenesis was analysed. In our system, the macrophage chemotactic activity of DBP and DBP-maf was very weak. Interestingly, an eluate from HHA adsorbed to normal human serum expressed significantly higher macrophage chemotactic activity than that from CHA (Fig. 5). The contribution of DBP to higher macrophage chemotactic activity of the eluate of HHA was not completely ruled out, but these findings strongly suggested that other factors included in the eluate of HHA caused induction of its higher chemotactic activity.

We precisely analysed the biological function of DBP and DBP-maf in osteoclastogenesis using an osteoclast coculture system 27. Supplementation of DBP to the culture medium revealed little effect on osteoclastogenesis (Fig. 7). In this coculture system, we used culture medium supplemented with 10% foetal bovine serum. According to previous studies, the mean serum concentration of DBP is about 200–500 μg/ml 38–39, and about 20–50 μg/ml DBP derived from foetal bovine serum was expected to be included in the culture medium. The stability and reactivity of DBP included in foetal bovine serum to mouse macrophages were unclear, but it was likely that supplementation of 0.01, 0.1 and 1 μg/ml human DBP had little effect in this culture system containing DBP derived from foetal bovine serum. In contrast, our results clearly showed that DBP-maf stimulated osteoclastogenesis (Fig. 7). Deglycosylation of DBP requires galactosidase and neuraminidase activities, and they were high in neutrophils and lymphocytes. Although the significance of preferential adsorption of DBP to HHA was not completely clarified in this study, there was a possibility that certain cells in the implanted region like neutrophils and lymphocytes expressed galactosidase and/or neuraminidase and synthesized a larger amount of DBP-maf in the region implanted with HHA than that implanted with CHA, and contributed to the potent osteoclast homing activity of HHA through the stimulation of osteoclastogenesis.

Among the list of components subjected to quantitative proteomic analysis, complements C3 and C4B adsorbed to HHA more than twice as much as CHA. These complements act as opsonins that bind with receptors on neutrophils and macrophages and promote their phagocytosis of coated cells 40. Interestingly, C3 was suggested to mediate the recruitment of osteoclasts to bone surface 41. In addition, C3 itself was suggested to promote osteoclastogenesis 42,43. Considering the results of these studies, C3 and C4B, especially C3, were thought to be candidates for proteins associated with the potent osteoclast homing activity of HHA.

The reason why the protein affinity is different between HHA and CHA remained unclear. It has been suggested that the HA crystal exposes different kinds of adsorption sites depending on the crystal face. Kawasaki *et al*. suggested that the *a*-face (the side surface of the hexagonal HA crystal) exposed positively charged sites 45. Rod-shaped particles in HHA were thought to dominantly expose the *a*-face compared with the *c*-face (the top and the bottom surfaces of the hexagonal HA crystal). On the other hand, particles in CHA were thought to expose the crystal face randomly. The difference in the exposed crystal faces between HHA and CHA was thought to affect the adsorption properties. Moreover, different pore structures between HHA and CHA may also have affected the affinity for proteins. In conclusion, serum proteins preferentially adsorbed to HHA were analysed and their relationship with the potent osteoclast homing activity of HHA in the implanted region was discussed. Quantitative proteomic analysis of eluates from HHA and CHA absorbed to normal human serum revealed preferential adsorption of DBP to HHA at the highest ratio compared with CHA. In addition, larger amounts of complements C3 and C4B were also detected in an eluate from HHA than from CHA. *In vitro* chemotactic analysis using chambers revealed that an eluate from HHA had significantly higher chemotactic activity than that from CHA, but the chemotactic activity of DBP and DBP-maf to macrophages was very weak. These results suggested that the higher chemotactic activity of an eluate from HHA than from CHA was induced not by DBP, but by other factors. Vitamin D-binding protein-maf stimulated osteoclastogenesis, and much of the DBP adsorbed to HHA might be deglycosylated and formed DBP-maf by certain cells with galactosidase and/or neuraminidase activity, like neutrophils and lymphocytes in the implanted region, which may be associated with the potent *in vivo* osteoclast homing activity of HHA in concert with the stimulation of osteoclastogenesis by C3 and opsonization promoted by C3 and C4B.
